# Evaluation of cylindrical micelles assembled from amphiphilic β-peptides as antigen delivery nanostructures†

**DOI:** 10.1039/d5na00166h

**Published:** 2025-03-20

**Authors:** Clément Martin, Mélanie Côté-Cyr, Phuong Trang Nguyen, Denis Archambault, Steve Bourgault

**Affiliations:** a Department of Chemistry, Université du Québec à Montréal C.P.8888, Succursale Centre-Ville Montréal H3C 3P8 Canada bourgault.steve@uqam.ca; b Quebec Network for Research on Protein Function, Engineering and Applications (PROTEO) Québec H3C 3P8 Canada; c The Swine and Poultry Infectious Diseases Research Centre (CRIPA) Saint-Hyacinthe J2S 2M2 Canada; d Department of Biological Sciences, Université du Québec C.P.8888, Succursale Centre-Ville Montréal H3C 3P8 Canada

## Abstract

Supramolecular nanostructures assembled from synthetic peptides constitute promising scaffolds for the delivery of antigens for vaccine development. Amphiphilic peptides and self-assembling cross-β-peptides have been shown to promote cellular uptake of antigenic epitopes by antigen-presenting cells, to stimulate the innate immune system and to induce a robust antigen-specific humoral immune response. In this study, we evaluated the use of cylindrical micelles assembled from the amphiphilic β-peptide C16V3A3K3 as a vaccine nanoplatform, combining the properties of cross-β-sheet fibrils and micelles. The ectodomain of the matrix 2 protein (M2e) of the influenza A virus was conjugated with a tetra-Gly linker at the C-terminus of C16V3A3K3. The chimeric peptide assembled into biocompatible unbranched filaments that exposed the antigen on the surface, and these filaments were readily internalized by dendritic cells and activated the toll-like receptor 2/6. These cylindrical micelles induced a robust M2e-specific humoral immune response upon intramuscular immunization in mice without the need for co-administration with adjuvants. Although this strong humoral response did not translate into protection against a lethal infection with the H1N1 influenza virus, these cylindrical micelles assembled from amphiphilic β-peptides expand the repertoire of self-adjuvanted nanostructures to enhance antibody production against peptide epitopes.

## Abstract 2

Cylindrical micelles assembled from synthetic amphiphilic β-peptides elicit a robust antigen-specific immune response against the grafted epitope.

## Graphical abstract

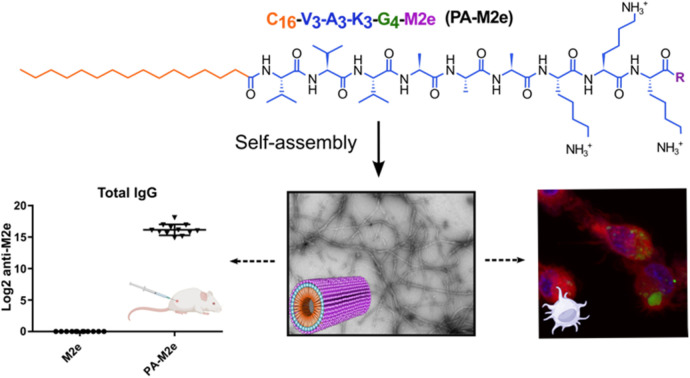

## 1 Introduction

Vaccination constitutes the most effective strategy to fight infectious diseases afflicting both humans and domesticated animals. Nonetheless, issues with safety and/or efficacy remain, notably with conventional vaccines based on inactivated or attenuated whole microorganisms.^1^ Over the last few decades, subunit vaccines composed of specific microbial antigens have been increasingly used due to their high safety, even though they tend to be weakly immunogenic and need to be administered with delivery vehicles and/or immunostimulatory agents to induce a robust and long-lasting antigen-specific immune response.^2^ Antigen delivery systems are known to increase the physicochemical stability of antigens, their biological half-life, their drainage to lymph nodes, and/or their internalization by antigen-presenting cells (APCs).^3–5^ Adjuvants act mainly by enhancing T and/or B cell responses through the engagement of specific components of the innate immune system.^5,6^ Interestingly, several proteinaceous nanoparticles used for the delivery of antigens in vaccines, including virus-like particles (VLPs) and peptide-based assemblies, have shown intrinsic adjuvant properties through different mechanisms, including activation of toll-like receptors (TLRs) and engagement of inflammasomes.^7–11^

Notably, supramolecular nanostructures obtained from the spontaneous self-assembly of synthetic peptides have shown potential for the development of subunit vaccines due to their biocompatibility, biodegradability, straightforward synthesis, easy characterization, and storage stability in lyophilized powder form.^1^ By varying the sequence of the peptide building block, a large array of mesoscopic architectures with tailored chemical and biological properties can be obtained.^12–14^ Through the attachment of an antigenic epitope to a self-assembling sequence, the resulting peptide nanostructures act as nanoplatforms for the presentation and delivery of antigenic determinants. For instance, several studies have shown that unbranched cross-β-sheet nanofibrils, which are obtained from the self-assembly of amyloid-like β-peptides, increase the antigen-specific immune response against the conjugated peptide epitopes.^9,15–19^ The cross-β-sheet quaternary organization of these fibrils is known to activate the heterodimeric TLR2/TLR6,^20–22^ as well as engage the cytosolic NLRP3 inflammasome,^23^ conferring self-adjuvanticity to these assemblies. Besides, amphiphilic peptides composed of a hydrophilic segment and a hydrophobic tail are known to self-assemble into micellar nanoparticles, which offer several advantages for antigen delivery. These spherical peptide micelles can protect the antigens from enzymatic degradation, enhance their bioavailability, and promote accumulation in the lymph nodes.^24,25^ Nanostructures assembled from amphiphilic peptides have shown immunostimulatory properties, including TLR2 activation and maturation of dendritic cells.^11,24^ Accordingly, it was observed in mice that peptide micelles induce an amplified antigen-specific humoral response against a peptide epitope grafted onto the surface.^24,26^

While cross-β-sheet nanofilaments and spherical amphiphilic micelles assembled from synthetic peptides have proven effective as epitope delivery systems, the use of cylindrical micelles based on the self-assembly of amphiphilic β-peptides as vaccine scaffolds has not been investigated so far. These biocompatible peptides have already been evaluated for different biomedical applications, including tissue regeneration^27,28^ and drug delivery.^28–31^ Amphiphilic β-peptides are composed of four domains: (i) a hydrophobic alkyl chain, (ii) a short hydrophobic sequence with a strong propensity to form intermolecular hydrogen bond ladders into cross-β-sheets, (iii) a highly charged region and (iv) a functional group that dictates the functionality of the nanostructure. Herein, we investigated the use of cylindrical micelles assembled from the C16V3A3K3 amphiphilic peptide (PA) to deliver antigenic epitopes. Towards this goal, the ectodomain of the matrix 2 protein (M2e) of the influenza A virus (IAV) was elongated at the C-terminus of PA by standard solid phase synthesis. The resulting elongated micelles engaged the heterodimeric TLR2–TLR6 and were avidly internalized by APCs, leading to a robust M2e-specific antibody response upon mice immunization. Taken together, this study identifies cylindrical micelles as a novel self-adjuvanted nanoplatform to enhance the production of epitope-specific antibodies.

## 2 Materials and methods

### 2.1 Synthesis, purification and self-assembly of peptides

Peptides were synthesized on solid support using Fmoc chemistry and a coupling method involving *N*,*N*-diisopropylethylamine as a base and 2-(6-chloro-1*H*-benzotriazol-1-yl)-1,1,3,3-tetramethylaminium hexafluorophosphate (HCTU), as previously described.^32^ Crude peptides were purified by HPLC using a C18 column with a linear gradient of acetonitrile in H_2_O/TFA (0.6% v/v). Fractions containing the desired peptide, validated by time-of-flight mass spectrometry (LC/MS-TOF), with a purity greater than 95% were pooled and lyophilized (Fig. S1–S3†). Peptides were dissolved at a concentration of 1.5 mM in a solution containing 20 mM Tris–HCl and 1.5 mM NaCl pH 7.4, and sonicated for 5 min. Self-assembly was carried out under constant rotary agitation at 40 rpm at room temperature for 96 h. For conjugation of the Alexa488 fluorophore, M2e and PA–M2e peptides were solubilized in H_2_O : acetonitrile (1 : 1) and the pH was adjusted to 9 with 4-methylmorpholine. Alexa488 succinimidyl ester was added at a molar ratio of 0.8 : 1 (Alexa488 : peptide), and the reaction mixture was incubated under constant rotary agitation at room temperature for 2 h before being purified by HPLC.

### 2.2 Fluorescence spectroscopy

Molecular self-assembly was monitored by measuring the extrinsic fluorescence using 8-anilinonaphthalene-1-sulfonic acid (ANS) and thioflavin T (ThT). At the desired time, peptide solutions were diluted in nanopure water to reach a final concentration of 50 μM. ANS and ThT were respectively added at final concentrations of 450 μM and 40 μM. ANS excitation was carried out at 355 nm, and the emission spectra were recorded from 385 to 585 nm. ThT emission spectra were recorded from 450 to 550 nm after excitation at 440 nm. Experiments were performed at least in triplicate with different peptide samples, and representative data are presented.

### 2.3 Circular dichroism spectroscopy

Peptide solutions were diluted in nanopure water to achieve a concentration of 50 μM. Circular dichroism (CD) analysis was conducted using a quartz cuvette with a 2-mm path length and spectra were recorded from 190 to 260 nm with a wavelength increment of 0.5 nm. The averaging time for each scan at each wavelength step was set to 10 s. Considering the presence of the lipid tail, the data are presented in mdeg rather than mean residue ellipticity (MRE). Experiments were performed at least in triplicate with different peptide samples, and representative data are shown.

### 2.4 Atomic force microscopy

Peptide assemblies were diluted in 1% (v/v) acetic acid to reach a final concentration of 25 μM and the resulting mixture was immediately applied to a freshly cleaved mica surface. After thorough washes with deionized water, samples were allowed to air dry for 24 h. Images were captured using a Veeco/Bruker Scan-Asyst multimode AFM in air mode, equipped with a silicon tip (tip radius of 2–12 nm and a constant force of 0.4 N m^−1^) mounted on a nitride lever, operating at a frequency of 0.9 Hz and 512 scans per minute. Images were analyzed using Gwyddion software.

### 2.5 Transmission electron microscopy

Peptide samples were diluted in H_2_O to a concentration of 50 μM and placed onto a glow-discharged, copper–carbon-coated 400 mesh grids. Negative staining was performed by applying 1.5% (w/v) uranyl formate for 1 min, followed by air drying for 24 h. Analyses were conducted using a FEI Tecnai G2 Spirit Twin TEM microscope operating at 120 kV and equipped with a AMT NanoSprint15 MK2 CMOS Camera.

### 2.6 Evaluation of epitope accessibility using ELISA

The accessibility of the M2e epitope on the peptide assemblies was assessed using an indirect ELISA. Briefly, high-binding 96-well plates were coated overnight at 4 °C with 2 μg of M2e peptide or an equivalent molar amount of PA–M2e assemblies. Plates were washed with PBS containing 0.05% Tween-20 (PBS-T) and blocked for 1 h at room temperature with PBS-T containing 1% (w/v) bovine serum albumin (BSA). After washing, the plates were incubated for 3 h at room temperature with a primary anti-M2 monoclonal antibody (14C2), diluted serially from 1 : 250 to 1 : 512 000. After extensive washing with PBS-T, rabbit anti-IgG conjugated to peroxidase was added at a dilution of 1 : 20 000, and the plates were incubated for 1 h at room temperature. Peroxidase activity was measured by adding 3,3′-5,5′-tetramethylbenzidine (TMB) and allowing the reaction to proceed for 20 min at room temperature before stopping it with H_2_SO_4_. Absorbance was measured at 450 nm, and the average absorbance of the blank was subtracted.

### 2.7 Cell viability

DC2.4 cells were cultured in RPMI-1640 supplemented with 10% (v/v) FBS, 2 mM l-glutamine, 0.1 mM non-essential amino acids, 25 mM HEPES buffer solution and 0.05 mM β-mercaptoethanol. DC2.4 cells were plated in 96-well plates at a density of 30 000 cells per well and incubated for 16 h with varying concentrations of PA or PA–M2e assemblies. After incubation, 50 μM resazurin was added, and the cells were incubated for an additional 3 h before measuring the absorbance at 570 nm. Cell viability (%) was determined by calculating the fluorescence ratio of the treated cells compared to control cells treated with the vehicle buffer (20 mM Tris–HCl, 1.5 mM NaCl pH 7.4).

### 2.8 Evaluation of TLR2/TLR6 activation

HEK-Blue hTLR2–TLR6 cells were maintained in DMEM enriched with 2 mM l-glutamine, 4.5 g L^−1^ glucose, 10% (v/v) fetal bovine serum, 100 μg mL^−1^ streptomycin, 100 U mL^−1^ penicillin, 100 μg mL^−1^ Normocin, and 1× HEK-Blue selection. Once the cell confluence reached around 60 to 80%, the cells were plated in a 96-well plate using HEK-Blue detection medium, mixed with equal volumes of peptide mixtures or buffer. Cells were seeded at a density of 280 000 cells per mL and 180 μL of the cell suspension was added per well. For measuring TLR2–TLR6 activity, peptide cylindrical micelles were incubated at concentrations ranging from 0.15 to 150 μM, with Pam2CSK4 used as a positive control at a concentration of 10 ng mL^−1^. After 16 h of incubation at 37 °C in 5% CO_2_, the absorbance was measured at 630 nm. The experiment was conducted at least three times in triplicate, and data were averaged and expressed as mean ± S.E.M.

### 2.9 Cellular uptake by dendritic cells

For flow cytometry analysis, DC2.4 cells were cultured in 24-well plates at a density of 50 000 cells per well. The following day, fluorescently labeled peptides were added to achieve an equimolar concentration of fluorophores. After 2 to 4 h of incubation at 37 °C, cells were thoroughly washed with ice-cold PBS and resuspended in PBS. Flow cytometry was performed using a CytoFLEX instrument, recording 10 000 synchronized events. Excitation wavelength was set at 488 nm, with emission detected at 530 nm. Collected data were analyzed using CytExpert software. This experiment was conducted at least three times in triplicate, and the results were averaged and presented as mean ± S.E.M. For confocal microscopy analysis, DC2.4 cells were cultured overnight in 8-well ibidi chamber slides at a density of 50 000 cells per well. The following day, after 4 h of incubation with fluorescently labeled peptides or micelles, the cells were thoroughly washed with ice-cold PBS and fixed for 10 min with 4% paraformaldehyde. Fixed cells were then stained with 50 ng mL^−1^ 4′,6-diamidino-2-phenylindole (DAPI) dichloride, and 1 U mL^−1^ Texas Red-X Phalloidin. Cells were washed with PBS, and a 10 mM Tris–HCl/glycerol solution (1 : 1 v/v) was added. Fluorescence images were captured using a Nikon A1R inverted confocal microscope with a 60× oil immersion lens, and the images were created through *Z*-axis stacking. Images were analyzed using Fiji ImageJ software, and representative images are presented.

### 2.10 Mice immunization

All protocols were approved by the Institutional Committee for Animal Protection and Use of the Université du Québec à Montréal in accordance with the regulations of the Canadian Council on Animal Care. Eight-week-old female BALB/c mice (8 mice per group) were intramuscularly immunized with 100 μL of soluble M2e antigen or cylindrical PA–M2e micelles (500 μM). Control mice were administered 100 μL of filtered sterilized 20 mM Tris–HCl, 1.5 mM NaCl at pH 7.4. Immunization was conducted three times, with a 2-week interval between each injection. Weights and clinical signs were monitored every day following each immunization. Blood samples were collected from the saphenous vein the day before each immunization, *i.e.* days 0, 14, and 28.

### 2.11 Determination of M2e-specific antibody titers

M2e-specific IgG antibody titers were assessed using indirect ELISA. High-binding 96-well plates were coated with 2 μg of M2e peptide per well, solubilized in 0.05 M sodium carbonate buffer at pH 9.6. Plates were washed thoroughly with PBS-T, blocked for 1 h with 1% (w/v) BSA, and then washed again with PBS-T. For titer determination, serial dilutions (1 : 2) of mouse sera, starting from 1 : 65 in PBS-T (1% BSA), were applied. After 3 h of incubation, plates were washed four times with PBS-T, followed by the addition of goat anti-mouse IgG conjugated to HRP (1 : 20 000), IgG1 (1 : 10 000), IgG2a (1 : 5000), IgG2b (1 : 5000), or IgG3 (1 : 5000) in PBS-T for 1 h. After washes, the TMB/peroxide substrate was added for 20 minutes, and the reaction was terminated by the addition of 1 N H_2_SO_4_. Absorbance was measured at 450 nm. Antibody titers were determined by plotting a regression curve [*y* = (*b* + *cx*)/(1 + *ax*)] of the different serum dilutions against optical density (OD). The final titers were defined as the highest dilution point that exhibited an optical density two times greater than that of the corresponding blanks, *i.e.* without serum.

### 2.12 Experimental challenge with H1N1 influenza A virus

This experiment was carried out under biosafety level 2 containment. Two weeks following the final immunization, mice were intranasally instilled with 5 × LD_50_ of the influenza A/Puerto Rico/8/1934 H1N1 virus in endotoxin-free PBS. Body weight and clinical signs were recorded twice daily, and clinical scores (Table S1†), ranging from 0 to 3, were monitored daily. Mice that achieved a clinical score of 3 or experienced a weight loss exceeding 20% of their initial weight were euthanized.

## 3 Results and discussion

### 3.1 Design, self-assembly and characterization of amphiphilic cylindrical micelles

**Fig. 1:**
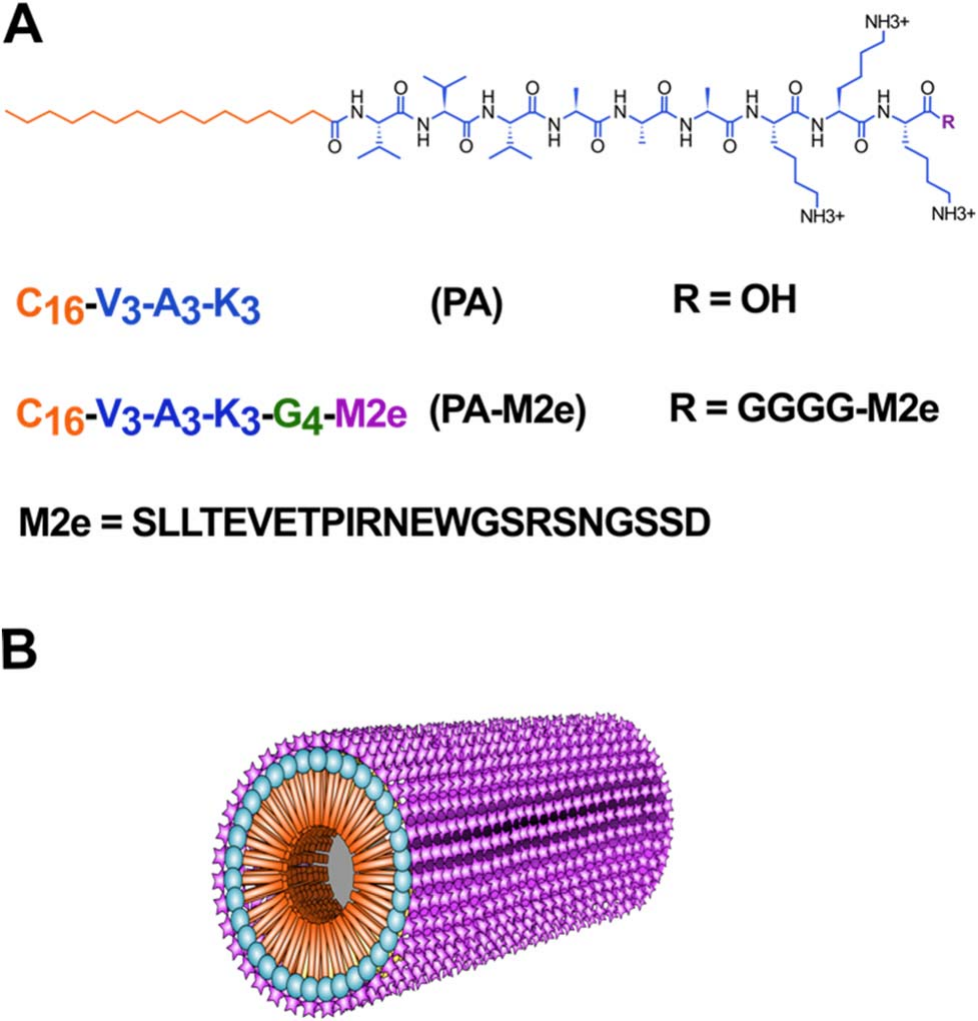
Design of cylindrical micelles based on the self-assembly of an amphiphilic β-peptide as an antigen delivery nanoplatform. (A) Molecular structure and sequences of peptides with the M2e epitope in purple, the GGGG linker in green, the self-assembling sequence in blue and the alkyl tail in orange. (B) Schematic representation of the cylindrical micelle with the M2e epitope represented on the surface.

To study the potential of cylindrical micelles assembled from amphiphilic β-peptides as antigen delivery nanoplatforms, the M2e epitope was fused to the C-terminus of the C16V3A3K3 self-assembling peptide (PA) *via* a flexible tetra-Gly spacer (Fig. 1). The M2e epitope, *i.e.* the ectodomain of the matrix 2 protein, is highly conserved among various strains of the influenza A virus, making M2e an attractive target for the development of universal flu vaccines.^33^ However, as a short and soluble peptide, the M2e epitope is poorly immunogenic when used alone, requiring the addition of adjuvants and/or conjugation to delivery nanosystems to induce a robust antigen-specific immune response. Herein, as supported by our previous studies using cross-β-sheet filaments,^16,19,23,34^ we hypothesized that the conjugation of M2e to PA will increase the antigen-specific humoral immune response by stabilizing the epitope, by promoting a depot effect at the immunization site, by facilitating internalization by APCs and/or by stimulating immune cells. The PA–M2e chimeric peptide was synthesized using standard solid phase synthesis and stored in lyophilized form to avoid premature self-assembly. Moreover, the two Cys at positions 17 and 19 of M2e were substituted with Ser to avoid oxidation and/or formation of disulfide bonds (Fig. 1), a modification that is known not to affect the immunogenicity of M2e and the affinity/selectivity of the resulting antibodies towards the M2 protein.^35^

**Fig. 2:**
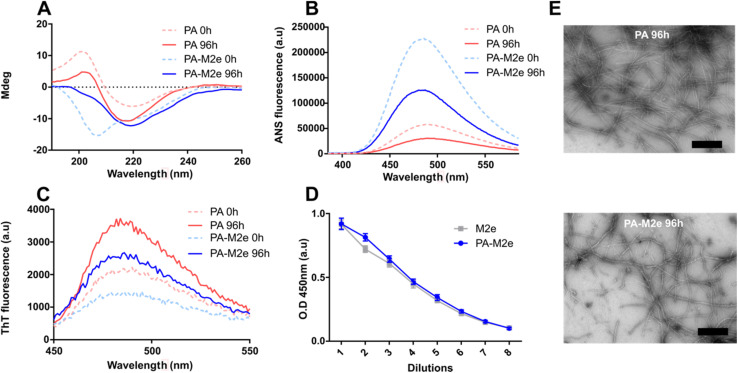
Self-assembly of amphiphilic β-peptides. (A) CD spectra of peptide solutions. (B) ANS fluorescence spectra of peptide solutions. (C) ThT fluorescence spectra of peptide solutions. (A–C) Analysis was performed immediately after resuspension (time = 0 h; dotted line) and after 96 h of incubation at room temperature under constant rotary agitation. (D) Accessibility of the M2e epitope on PA–M2e assemblies using indirect ELISA. (E) Representative negative-stain TEM images of PA and PA–M2e after 96 h of incubation under constant rotary agitation. The scale bar is 500 nm.

Peptide self-assembly was initiated by dispersing the lyophilized peptide at a concentration of 1.5 mM in 20 mM Tris–HCl, pH 7.4, supplemented with 1.5 mM NaCl. Self-assembly performed under constant rotary agitation (40 rpm) was monitored by circular dichroism (CD) spectroscopy, thioflavin T (ThT) fluorescence, and 1-anilino-8-naphthalenesulfonate (ANS) fluorescence. Immediately after solubilisation, *i.e.*, at time 0 h, the CD spectrum of PA showed a minimum around 218 nm, indicative of a β-sheet-rich conformation, while the CD spectrum of PA–M2e indicated a random coil conformation, which progressively shifted to a β-sheet signal upon 96 h of incubation (Fig. 2A and S4†). Thus, the presence of the M2e antigen at the C-terminus of PA slowed down the self-assembly into a β-sheet-rich conformation. According to CD analyses (Fig. S4†), PA–M2e self-assembly reached equilibrium after 96 h and this incubation time was selected to prepare the nanovaccine. Both peptides were characterized by a decrease in ANS fluorescence upon 96 h of incubation (Fig. 2B). ANS is a probe that emits fluorescence upon binding to hydrophobic interfaces, although it tends to remain on the surface of nanostructures without penetrating their core.^36^ Thus, the decrease in the ANS signal during assembly could be associated with the fact that the hydrophobic tails become more buried into the micelles upon elongation. The formation of the cross-β-sheet quaternary structure involving the V_3_A_3_ region was analyzed using ThT fluorescence. This benzothiazole dye shows a significant increase in its fluorescence quantum yield upon binding to cross-β-sheet quaternary structures.^37,38^ For both peptides, an increase in the ThT fluorescence signal was recorded between 0 and 96 h of incubation, indicative of the formation of cross-β organization during assembly (Fig. 2C). The accessibility of the M2e epitope upon assembly was evaluated using ELISA. Results showed that the ELISA signal of the assemblies was comparable to the signal observed for the soluble M2e peptide, indicating high accessibility of the antigen on the surface of PA–M2e assemblies (Fig. 2D). The nanostructures obtained after 96 h of incubation were observed by transmission electron microscopy (TEM) (Fig. 2E) and atomic force microscopy (AFM) (Fig. S5†). The obtained images confirmed the formation of long and unbranched filaments, consistent with cylindrical micelles previously reported,^39,40^ with the addition of the M2e epitope not affecting significantly the morphology nor the size of the nanostructures (Fig. 2E). In fact, quantification and distribution of length of the filaments from AFM images revealed a similar average length for both peptide assemblies, with an average of 0.472 ± 0.021 μm for PA–M2e and 0.419 ± 0.023 μm for PA (Fig. S5†).

### 3.2 Cytocompatible M2e-conjugated filaments are taken up by dendritic cells and activate the heterodimeric TLR2/TLR6

**Fig. 3:**
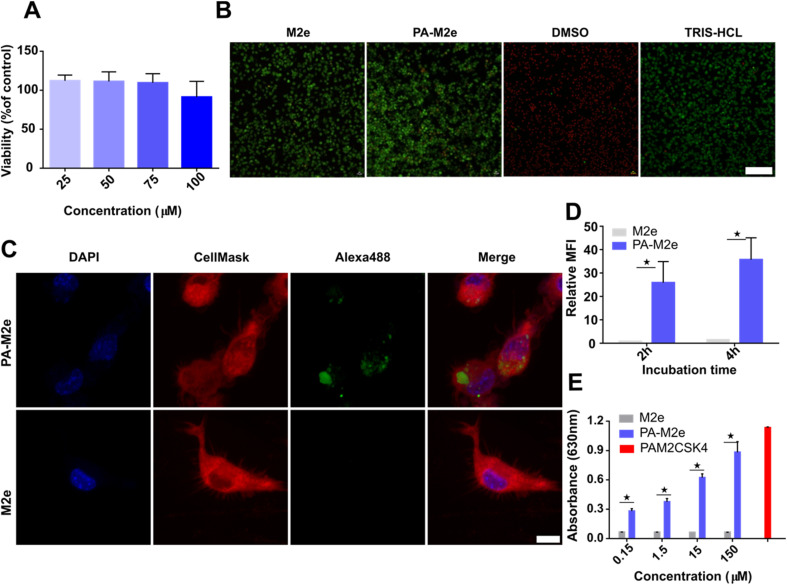
Biological characterization of M2e–PA amphiphilic peptide filaments. (A) Viability of DC2.4 cells after treatment with peptide. Cells were exposed to cylindrical micelles for 16 h, and metabolic activity was assessed by resazurin reduction. (B) Representative fluorescence microscopy images showing the distribution of live (green) and dead (red) DC2.4 cells after treatment with 100 μM peptide assemblies for 16 h. The scale bar is 100 μm. (C) Representative confocal microscopy images of DC2.4 cells incubated with 100 μM of Alexa488-labeled assemblies and molar equivalent of monomeric labelled M2e for 4 h at 37 °C. The scale bar is 10 μm. (D) Mean fluorescence intensity (MFI) of DC2.4 cells incubated for 2 and 4 h with 100 μM of Alexa488-labeled assemblies or molar equivalent of monomeric M2e. MFI values are expressed relative to PBS-treated cells. Statistical significance was determined by a multiple *t*-test (*P < 0.05). (E) Activation of TLR2–TLR6 by PA–M2e assemblies. HEK-Blue cells expressing hTLR2–TLR6 were incubated with nanofilaments for 16 h, and activation was measured using the SEAP reporter. Statistical significance was assessed by a multiple *t*-test (*P < 0.05).

Micellar filaments assembled from C16V3A3K3 amphiphilic peptides are known for their high biocompatibility, prompting their usage for different biomedical applications.^41^ Nonetheless, before moving forward with mice immunization, we evaluated whether the addition of the M2e peptide led to any unexpected cytotoxicity. Dendritic DC2.4 cells were incubated with increasing concentrations of pre-assembled micellar assemblies for 16 h, and metabolic activity was assessed by resazurin reduction. PA–M2e assemblies did not significantly decrease cell viability, even at the highest concentration evaluated, *i.e.*, 100 μM (Fig. 3A). A live–dead cell assay using confocal microscopy further confirmed the biocompatibility of PA–M2e assemblies upon treatment of dendritic cells (Fig. 3B).

Dendritic cells play a critical role in bridging innate and adaptive immunity and are important APCs associated with the establishment of immunological memory.^42^ Thus, the uptake of antigens by dendritic cells constitutes a key step in the formation of a robust and long-lasting immune response. Accordingly, we evaluated the internalization of cylindrical micelles decorated with the M2e peptide by dendritic cells by confocal fluorescence microscopy and flow cytometry. Fluorescent cylindrical micelles were obtained by co-assembling fluorescently labelled PA–M2e peptide (0.05 eq.) with unlabelled PA–M2e, leading to the formation of unbranched filaments characterized by a β-sheet conformation (Fig. S6†). Dendritic cells were incubated for 4 h in the presence of fluorescent PA–M2e assemblies and confocal microscopy imaging revealed green fluorescence puncta within the cytoplasm (Fig. 3C). In sharp contrast, no significant fluorescence could be detected within dendritic cells after treatment with an equivalent molar amount of soluble M2e labeled with the Alexa488 fluorophore. Internalization by DC2.4 cells was further evaluated by flow cytometry, and the results confirmed that the M2e-functionalized assemblies were significantly more internalized compared to their monomeric counterpart (Fig. 3D). As previously reported for cross-β-sheet fibrils^16,20,21,23^ and spherical amphiphilic micelles,^24,43^ peptide nanostructures can activate receptors of the innate immune systems, notably the heterodimeric TLR2/TLR6 membrane receptor. Accordingly, we evaluated the activation of TLR2/TLR6 by PA–M2e assemblies using HEK-Blue hTLR2–TLR6 cells, which express TLR2–TLR6 along with an NF-κB-inducible SEAP reporter gene. The results showed that pre-assembled PA–M2e nanofilaments activated the hTLR2–6 signaling pathway in a concentration-dependent manner, while the soluble M2e peptide did not lead to any significant NF-κB activation (Fig. 3E).

### 3.3 PA–M2e cylindrical micelles induce a robust antigen-specific response in mice

**Fig. 4:**
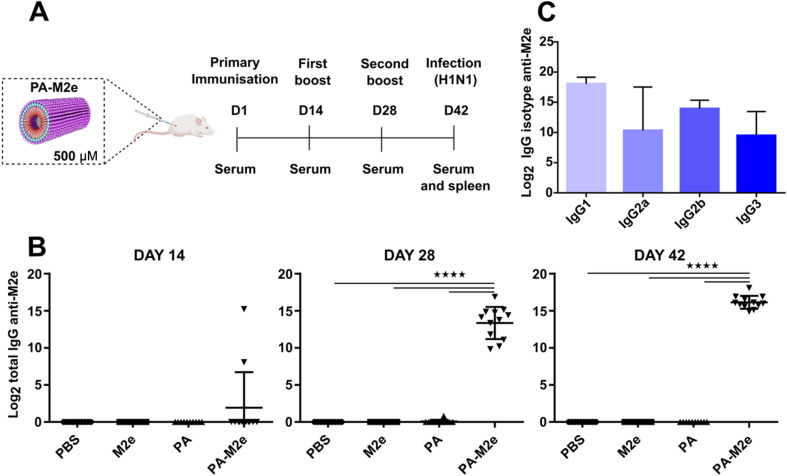
Cylindric micelles induce a robust production of antigen-specific antibodies upon mice immunization. (A) Immunization, sampling, and infection schedule. (B) Total IgG titers from mouse serum after the first, second, and third immunizations, measured using indirect anti-M2e ELISA. (C) Serum IgG isotypes determined using indirect ELISA. Data represent the mean ± S.E.M. and statistical significance between groups was assessed using one-way ANOVA with Tukey's multiple-comparison test (****P < 0.0001).

After confirming that the PA–M2e peptide assembles into biocompatible filamentous nanostructures displaying the M2e antigen on their surface, we evaluated the M2e-specific antibody response and the potential protection against an experimental viral challenge in mice. Female BALB/c mice were immunized three times intramuscularly at 14-day intervals, and sera were collected one day before each immunization (Fig. 4A). Upon immunization, none of the mice experienced weight loss (Fig. S7†) or developed any noticeable clinical sign (data not shown), indicative of the innocuity of the vaccine formulation. Sera from mice immunized with the soluble M2e antigen revealed no M2e-specific IgG antibodies, even after the second boost (Fig. 4B), consistent with the low immunogenicity of soluble peptides.^33^ In contrast, after the second immunization, all PA–M2e immunized mice produced high levels of anti-M2e IgG. Notably, two mice immunized with PA–M2e filaments showed significant levels of M2e-specific antibodies after the primary immunization. The second boost further increased the M2e-specific antigen response, raising the average antibody titer up to 16 log_2_ (Fig. 4B). Moreover, mice immunized with the PA–M2e assemblies produced significant levels of M2e-specific IgG1, IgG2a, IgG2b, and IgG3 isotypes (Fig. 4C), suggestive of a balanced Th1/Th2 immune response.^44,45^

**Fig. 5:**
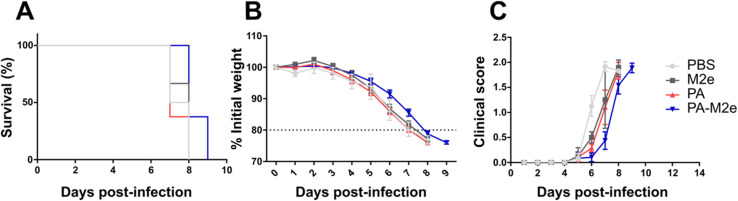
Intramuscular immunization with PA–M2e assemblies does not protect mice against influenza A H1N1 virus. (A) Survival, (B) weight loss and (C) clinical score of immunized mice following infection with 5 × LD_50_ of influenza A/Puerto Rico/8/1934 virus.

Despite a robust and balanced antigen-specific antibody response in mice immunized with PA–M2e in the absence of a supplemented adjuvant, none of them survived a lethal infection with 5 × LD_50_ of influenza A H1N1 virus (Fig. 5). These results contrast with observations reported in the literature, where several antigen delivery nanoplatforms demonstrated protection in immunized mice against fatal influenza infections *via* an M2e-specific immune response.^16,46^ This absence of protection was unexpected, considering that previous studies have indicated that a robust M2e-specific humoral response is sufficient to provide protection, at least partial, against H1N1 infection.^47,48^ It is known that IgG2a antibodies play a crucial role in inducing ADCC and/or ADCP mechanisms, which are essential for anti-M2e protection.^47–49^ Our results revealed an apparent lower IgG2a production compared to IgG1 and IgG2b (Fig. 4C), which could explain the lack of observed protection. Nevertheless, an in-depth evaluation of the ability of IgG2a, induced by the PA–M2e nanovaccine, to trigger ADCC and ADCP mechanisms would be critical.

Considering that cytotoxic T cells also play an important role in combating influenza virus infection,^49,50^ we characterized activated lymphocyte populations in the spleen of immunized mice by exposing isolated splenocytes to the M2e antigen and measuring the production of IFN-γ and IL-4. Using ELISpot, PA–M2e-immunized mice did not produce significantly more splenocytes secreting IFN-γ or IL-4 than mice that received the control PBS vehicle alone (Fig. S8 and S9†). These observations indicate the absence of M2e-responsive T cells in the spleen of immunized mice, which could possibly explain the lack of protection observed despite high levels of anti-M2e antibodies. The mechanisms underlying M2e-mediated protection appear to be diverse. For instance, it has been demonstrated that the presence of Th17 cells and alveolar macrophages alone can protect against lethal influenza infections, even in the absence of a humoral response.^51^ Further analysis to characterize the presence and activation of these cells in response to the M2e antigen, particularly through the secretion of specific cytokines, could help clarify the unexpected results of this study.

## 4 Conclusion

Herein, we report that nanostructures assembled from the amphiphilic β-peptide C16V3A3K3 are interesting candidates as antigen delivery nanoplatforms due to their high biocompatibility, ability to be functionalized with peptide epitopes, intrinsic immunostimulatory properties, and capacity to promote a robust antigen-specific systemic humoral response in mice. It is noteworthy that the isotype profile of the antibody response obtained by immunizing mice with PA–M2e was strong and diverse, with the presence of IgG1, IgG2a, IgG2b, and IgG3. However, this robust antibody response was not sufficient to protect immunized mice infected with 5 × LD_50_ of the H1N1 influenza A virus. Notably, data regarding the cellular response profile in splenocytes following immunization showed an absence of cellular immunity, which could explain the lack of protection against an experimental challenge. Further studies will be needed to better characterize the immune responses following immunization with PA–M2e and to clarify the mechanisms leading to immunological protection. Moreover, as the length, and size of the antigen delivery nanosystems are known to significantly affect the immune responses,^7^ it will be critical to further investigate how the macroscopic heterogenicity of the cylindrical micelle formulation affects its immunogenicity. The elucidation of the relationships between the length of the peptide nanofilaments and their immunomodulatory properties remains particularly important. Although this study did not demonstrate protection against influenza infection, antigen-specific antibodies remain key effectors in combating many infectious agents, such as rotavirus causing neonatal diarrhea,^52^ and human respiratory syncytial virus.^53^ The nanostructures based on amphiphilic β-peptides described here remain promising for vaccine applications. Micellar cylindrical micelles could address potential limitations of amyloid cross-β fibrils, *i.e.* their potential prion-like effect and cross-aggregation of endogenous proteins, and of spherical micelles, *i.e.* low physicochemical and metabolic stability. Moreover, fully synthetic peptide nanoplatforms offer notable advantages, including storage stability, versatility and biocompatibility. It would be feasible to co-assemble two PA molecules functionalized differently, *i.e.* one carrying the M2e antigen and the other one with an adjuvant such as CpG, known for inducing a strong cellular response.^16,54^ Such an approach could stimulate robust humoral and cellular immune responses, thereby providing effective protection against the influenza A virus.

## Data availability

When applicable, the data supporting this article have been included as part of the ESI.†

## Conflicts of interest

There are no conflicts to declare.

## Supplementary Material

NA-OLF-D5NA00166H-s001

## References

[cit1] Gebre M. S., Brito L. A., Tostanoski L. H., Edwards D. K., Carfi A., Barouch D. H. (2021). Cell.

[cit2] Vartak A., Sucheck S. J. (2016). Vaccines.

[cit3] Gregory A. E., Titball R., Williamson D. (2013). Front. Cell. Infect. Microbiol..

[cit4] Al-Halifa S., Babych M., Zottig X., Archambault D., Bourgault S. (2019). Pept. Sci..

[cit5] Chatzikleanthous D., O'Hagan D. T., Adamo R. (2021). Mol. Pharmaceutics.

[cit6] Coffman R. L., Sher A., Seder R. A. (2010). Immunity.

[cit7] Zottig X., Côté-Cyr M., Arpin D., Archambault D., Bourgault S. (2020). Nanomaterials.

[cit8] Zhao G., Chandrudu S., Skwarczynski M., Toth I. (2017). Eur. Polym. J..

[cit9] Rudra J. S., Tian Y. F., Jung J. P., Collier J. H. (2010). Proc. Natl. Acad. Sci. U. S. A..

[cit10] Eskandari S., Guerin T., Toth I., Stephenson R. J. (2017). Adv. Drug Delivery Rev..

[cit11] Castelletto V., Kirkham S., Hamley I. W., Kowalczyk R., Rabe M., Reza M., Ruokolainen J. (2016). Biomacromolecules.

[cit12] Fan J., Toth I., Stephenson R. J. (2024). Immuno.

[cit13] Gazit E. (2007). Chem. Soc. Rev..

[cit14] Fan T., Yu X., Shen B., Sun L. (2017). J. Nanomater..

[cit15] Babych M., Bertheau-Mailhot G., Zottig X., Dion J., Gauthier L., Archambault D., Bourgault S. (2018). Nanoscale.

[cit16] Bricha S., Côté-Cyr M., Tremblay T., Nguyen P. T., St-Louis P., Giguère D., Archambault D., Bourgault S. (2023). ACS Infect. Dis..

[cit17] Rudra J. S., Mishra S., Chong A. S., Mitchell R. A., Nardin E. H., Nussenzweig V., Collier J. H. (2012). Biomaterials.

[cit18] Chen J.-L., Fries C. N., Berendam S. J., Rodgers N. S., Roe E. F., Wu Y., Li S. H., Jain R., Watts B., Eudailey J. (2022). Sci. Adv..

[cit19] Zottig X., Al-Halifa S., Côté-Cyr M., Calzas C., Le Goffic R., Chevalier C., Archambault D., Bourgault S. (2021). Biomaterials.

[cit20] Al-Halifa S., Zottig X., Babych M., Côté-Cyr M., Bourgault S., Archambault D. (2020). Nanomaterials.

[cit21] Kihal N., Archambault M.-J., Babych M., Nazemi A., Bourgault S. (2024). Soft Matter.

[cit22] Tükel Ç., Wilson R. P., Nishimori J. H., Pezeshki M., Chromy B. A., Bäumler A. J. (2009). Cell Host Microbe.

[cit23] Lamontagne F., Arpin D., Côté-Cyr M., Khatri V., St-Louis P., Gauthier L., Archambault D., Bourgault S. (2023). Adv. Healthcare Mater..

[cit24] Trent A., Ulery B. D., Black M. J., Barrett J. C., Liang S., Kostenko Y., David N. A., Tirrell M. V. (2015). AAPS J..

[cit25] Barrett J. C., Ulery B. D., Trent A., Liang S., David N. A., Tirrell M. V. (2017). ACS Biomater. Sci. Eng..

[cit26] Zhang R., Smith J. D., Allen B. N., Kramer J. S., Schauflinger M., Ulery B. D. (2018). ACS Biomater. Sci. Eng..

[cit27] Webber M. J., Kessler J., Stupp S. (2010). J. Intern. Med..

[cit28] Webber M. J., Berns E. J., Stupp S. I. (2013). Isr. J. Chem..

[cit29] Zhao C., Chen H., Wang F., Zhang X. (2021). Colloids Surf., B.

[cit30] Fuertes-Llanos M. A., Gómara M. J., Haro I., Sánchez-López E. (2024). Curr. Med. Chem..

[cit31] Habibi N., Kamaly N., Memic A., Shafiee H. (2016). Nano Today.

[cit32] Tchoumi Neree A., Nguyen P. T., Chatenet D., Fournier A., Bourgault S. (2014). FEBS Lett..

[cit33] Deng L., Cho K. J., Fiers W., Saelens X. (2015). Vaccines.

[cit34] St-Louis P., Martin C., Khatri V., Bourgault S., Archambault D. (2024). Vaccine.

[cit35] Nguyen P. T., Zottig X., Sebastiao M., Arnold A. A., Marcotte I., Bourgault S. (2021). Commun. Biol..

[cit36] Liu X., Gao H., Huang F., Pei X., An Y., Zhang Z., Shi L. (2013). Polymer.

[cit37] Naiki H., Higuchi K., Hosokawa M., Takeda T. (1989). Anal. Biochem..

[cit38] Sebastiao M., Quittot N., Bourgault S. (2017). Anal. Biochem..

[cit39] Hartgerink J. D., Beniash E., Stupp S. I. (2002). Proc. Natl. Acad. Sci. U. S. A..

[cit40] Hartgerink J. D., Beniash E., Stupp S. I. (2001). Science.

[cit41] Singh R., Sharma S., Kautu A., Joshi K. B. (2024). Chem. Commun..

[cit42] Cabeza-Cabrerizo M., Cardoso A., Minutti C. M., Pereira da Costa M., Reis e Sousa C. (2021). Annu. Rev. Immunol..

[cit43] Wen Y., Collier J. H. (2015). Curr. Opin. Immunol..

[cit44] Huber V. C., McKeon R. M., Brackin M. N., Miller L. A., Keating R., Brown S. A., Makarova N., Perez D. R., MacDonald G. H., McCullers J. A. (2006). Clin. Vaccine Immunol..

[cit45] Hovden A. O., Cox R., Haaheim L. (2005). Scand. J. Immunol..

[cit46] Wang Q., Zhang Y., Zou P., Wang M., Fu W., She J., Song Z., Xu J., Huang J., Wu F. (2020). Front. Microbiol..

[cit47] Sun T., Wang Y., Zou P., Wang Q., Liu J., Liu W., Huang J., Wu F. (2023). J. Med. Virol..

[cit48] El Bakkouri K., Descamps F., De Filette M., Smet A., Festjens E., Birkett A., Van Rooijen N., Verbeek S., Fiers W., Saelens X. (2011). J. Immunol..

[cit49] Van den Hoecke S., Ehrhardt K., Kolpe A., El Bakkouri K., Deng L., Grootaert H., Schoonooghe S., Smet A., Bentahir M., Roose K. (2017). J. Virol..

[cit50] Zykova A. A., Blokhina E. A., Stepanova L. A., Shuklina M. A., Tsybalova L. M., Kuprianov V. V., Ravin N. V. (2022). Nanomed. Nanotechnol. Biol. Med..

[cit51] Eliasson D., Omokanye A., Schön K., Wenzel U., Bernasconi V., Bemark M., Kolpe A., El Bakkouri K., Ysenbaert T., Deng L. (2018). Mucosal Immunol..

[cit52] Ward R. L. (1996). J. Infect. Dis..

[cit53] Galvez N., Soto J. A., Bueno S. M., Kalergis A. M. (2020). J. Immunol..

[cit54] Kocabas B. B., Almacioglu K., Bulut E. A., Gucluler G., Tincer G., Bayik D., Gursel M., Gursel I. (2020). J. Controlled Release.

