# Supplementary material for: Evaluation of cylindrical micelles assembled from amphiphilic β-peptides as antigen delivery nanostructures
Source: Nanoscale Adv. 2025 Mar 20;7(10):2979–87. doi: 10.1039/d5na00166h (PMC11960782; doi:10.1039/d5na00166h)
Supplement: NA-007-D5NA00166H-s001 [file NA-007-D5NA00166H-s001.pdf]

Supplementary Information (SI) for Nanoscale Advances.
This journal is © The Royal Society of Chemistry 2025

Evaluation of cylindrical micelles assembled from amphiphilic β-peptides as antigen delivery nanostructures

Clément Martin[1,2,3,4], Mélanie Côté-Cyr[1,2,3], Phuong Trang Nguyen[1,2,3], Denis Archambault[3,4,*], Steve Bourgault[1,2,4,*]

[1]*Department of Chemistry, Université du Québec à Montréal. C.P.8888, Succursale Centre-Ville, Montréal, H3C 3P8, Canada*
[2]*Quebec Network for Research on Protein Function, Engineering and Applications (PROTEO), Québec, H3C 3P8, Canada*
[3]*The Swine and Poultry Infectious Diseases Research Centre (CRIPA), Saint-Hyacinthe, J2S 2M2 Canada*
[4]*Faculty of Sciences, Université du Québec à Montréal. C.P.8888, Succursale Centre-Ville, Montréal, H3C 3P8, Canada*

<u>Table of contents</u>

Figure S1 HPLC chromatogram and MS-TOF analysis of PA-M2e………………….. 3

Figure S2 HPLC chromatogram and MS-TOF analysis of PA……………………….. 4

Figure S3 HPLC chromatogram and MS-TOF analysis of M2e………………………. 5

Figure S4 CD spectra of PA-M2e and PA ……………………………………………. 6

Figure S5 Representative AFM and size distribution of PA-M2e and PA…………….. 7

Figure S6 CD spectra and representative AFM of PA-M2e-alexa488………………... 8

Figure S7 Weight loss of mice after immunization………..…………………………... 9

Figure S8 Elispot IFN-γ………………………………………………………………. 10

Figure S9 Elispot IL-4……………………………………………………………… 11

Table S1 Scale for clinical symptoms of influenza infection………………………….. 12

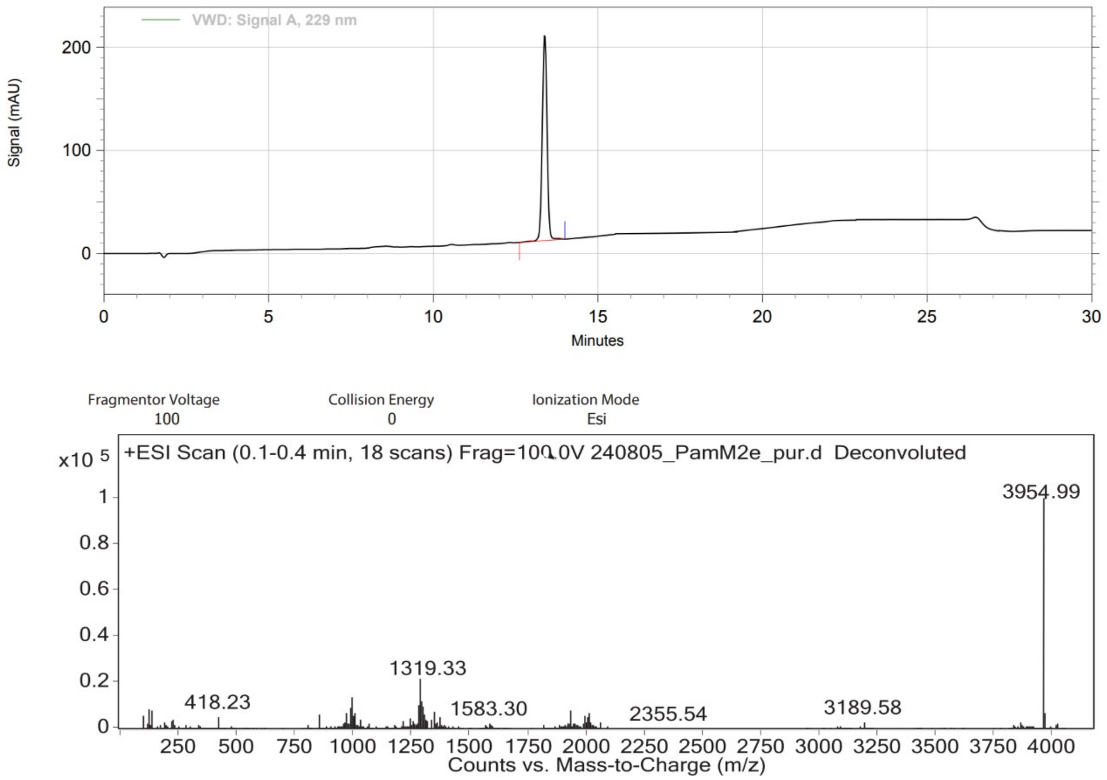

Figure S1 HPLC chromatogram and MS-TOF spectra of PA-M2e. HPLC analysis was performed using a C18 column with a linear gradient of 20% to 60% acetonitrile in H_2O/TFA (0.06%) over 20 minutes, while absorbance was monitored at 229 nm. Mass spectrometry analysis was conducted using an ESI-TOF mass spectrometer.

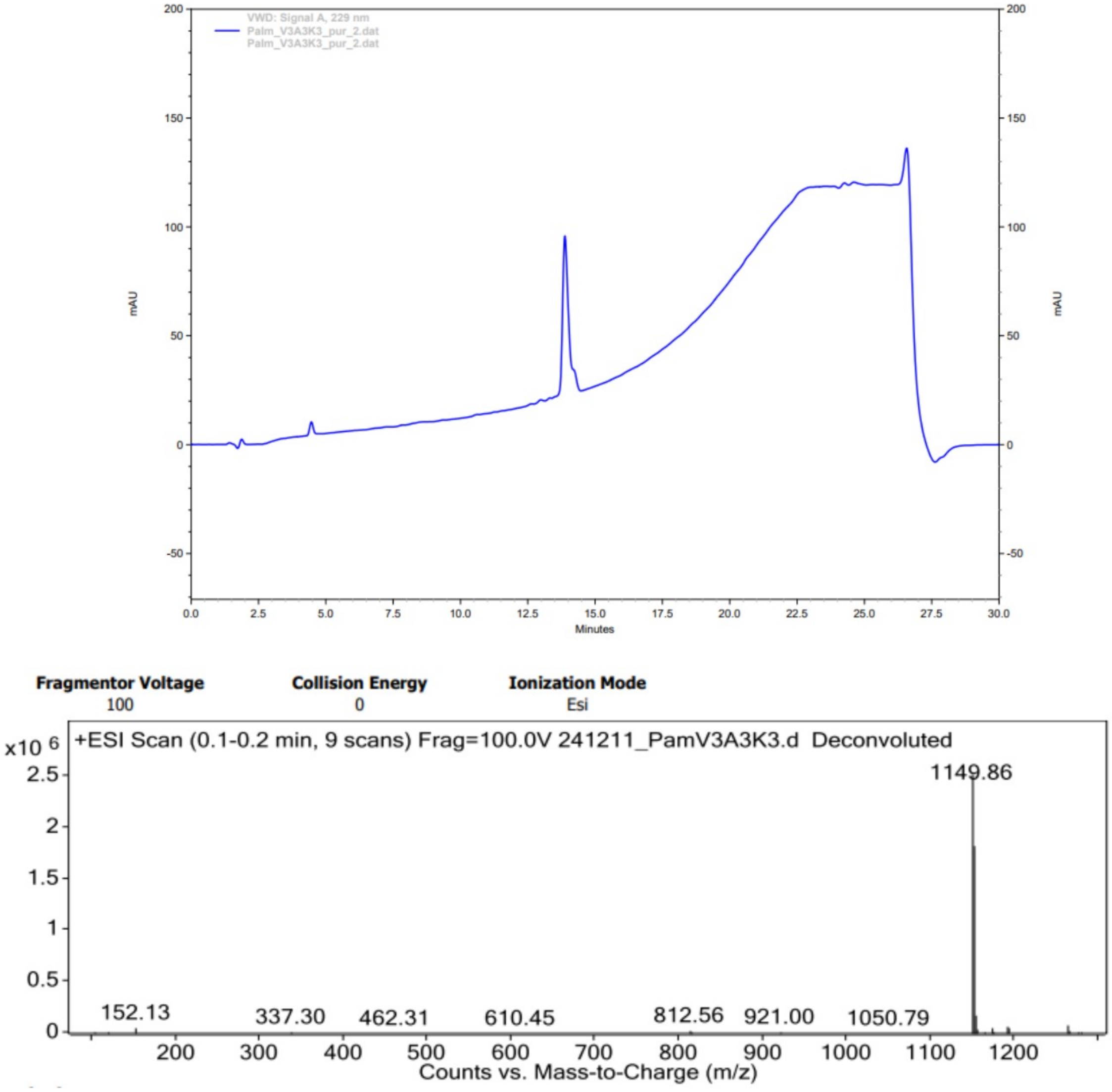

Figure S2 HPLC chromatogram and MS-TOF spectra of PA. HPLC analysis was performed using a C18 column with a linear gradient of 20% to 60% acetonitrile in H_2O/TFA (0.06%) over 20 minutes, while absorbance was monitored at 229 nm. Mass spectrometry analysis was conducted using an ESI-TOF mass spectrometer.

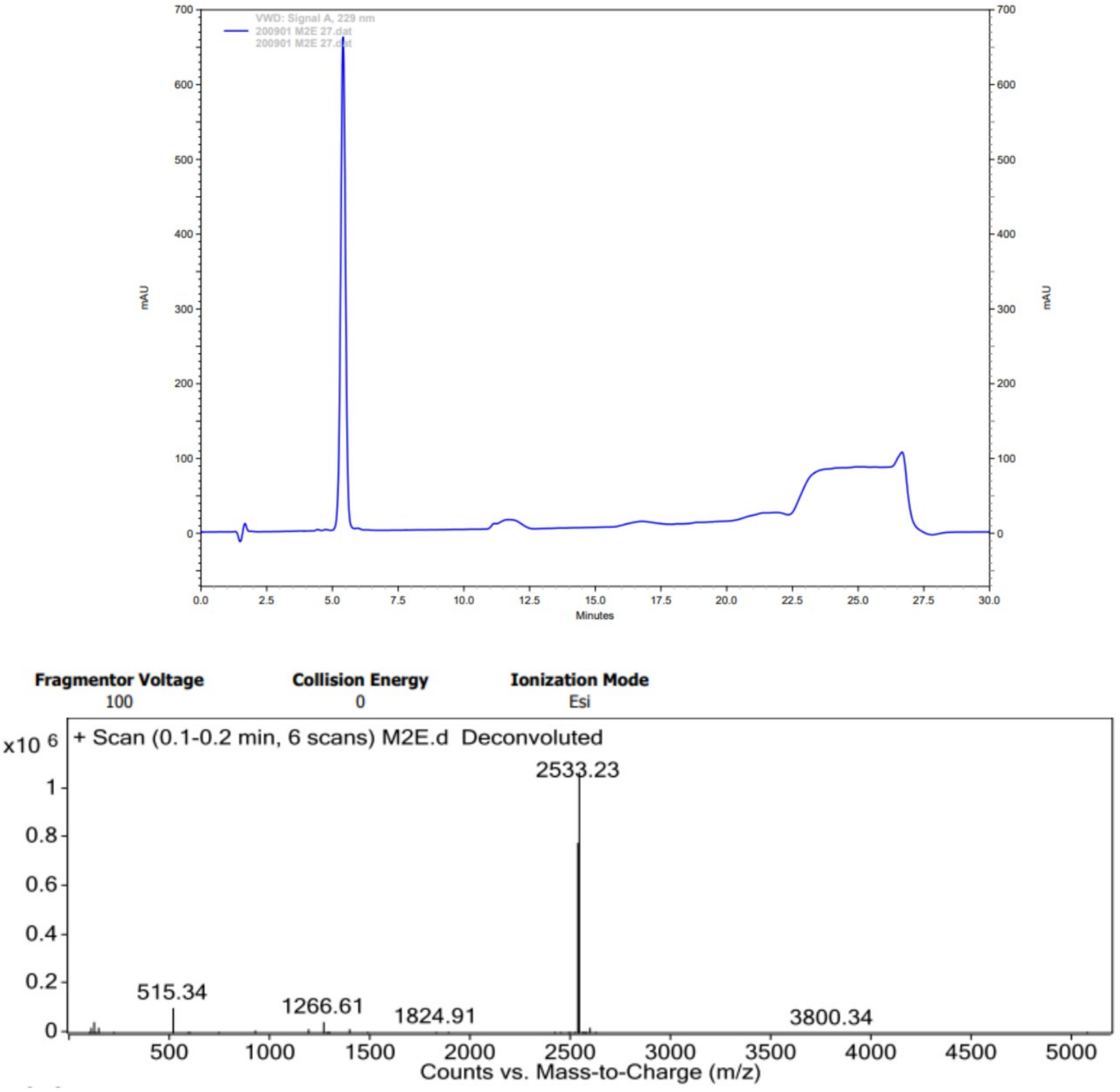

Figure S3 HPLC chromatogram and MS-TOF spectra of M2e. HPLC analysis was performed using a C18 column with a linear gradient of 20% to 60% acetonitrile in H2O/TFA (0.06%) over 20 minutes, while absorbance was monitored at 229 nm. Mass spectrometric analysis was conducted using an ESI-TOF mass spectrometer.

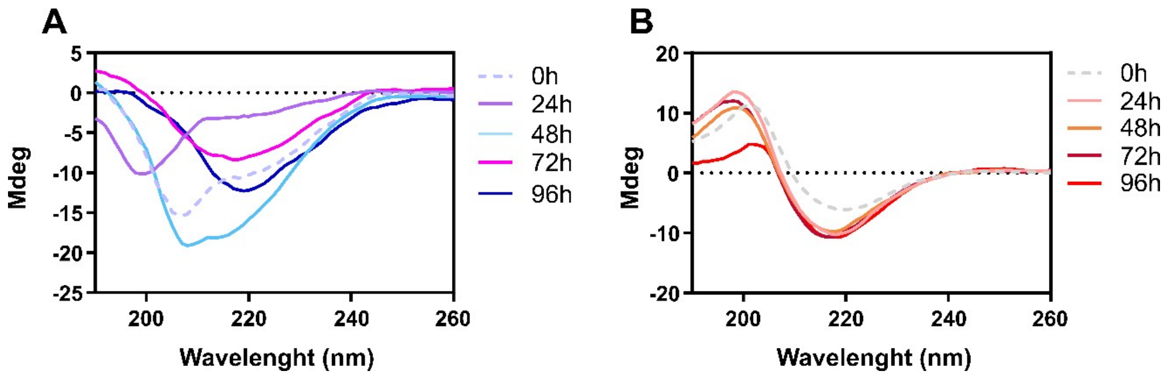

Figure S4 CD spectra of PA-M2e (A) and PA (B) over incubation time.

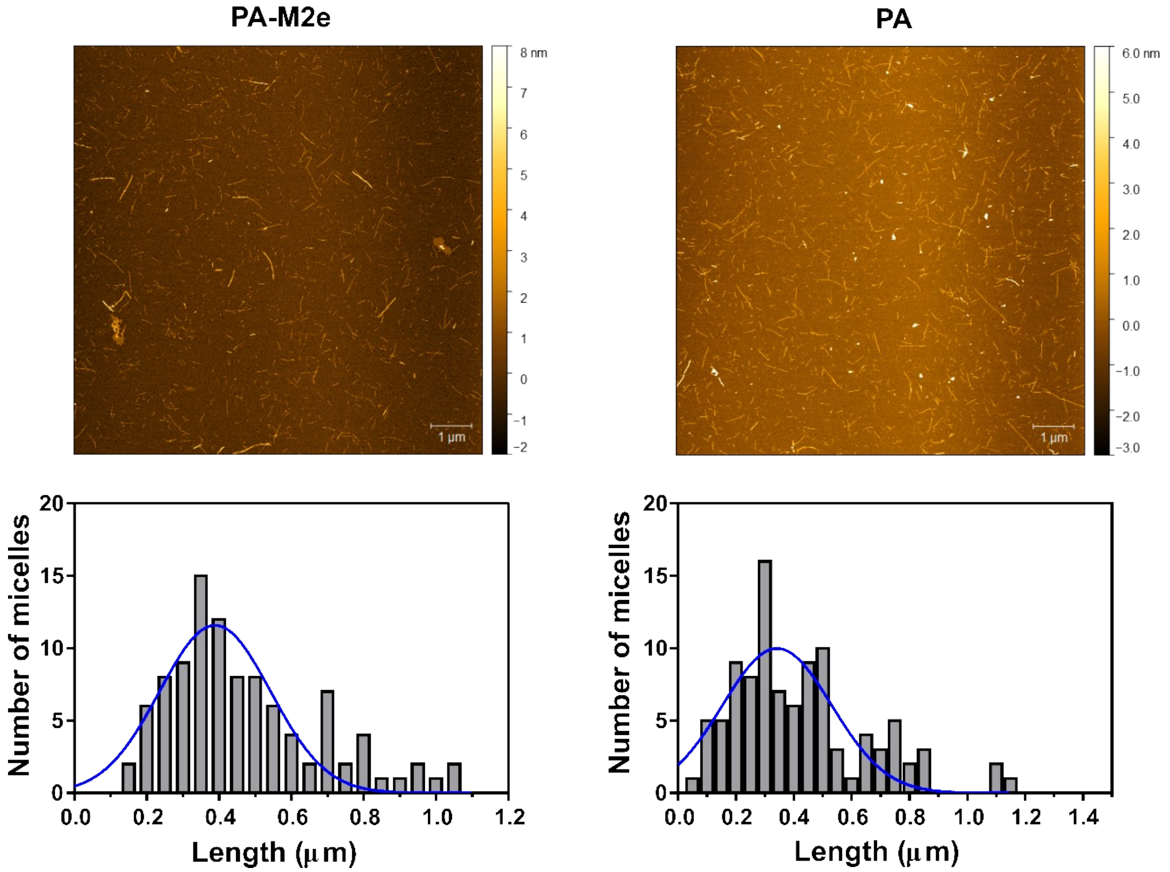

Figure S5 Representative of atomic force microscopy and size distribution of PA-M2e and PA. Assemblies were obtained after 96h incubation under constant agitation at RT. Scale bar is 1 μm.

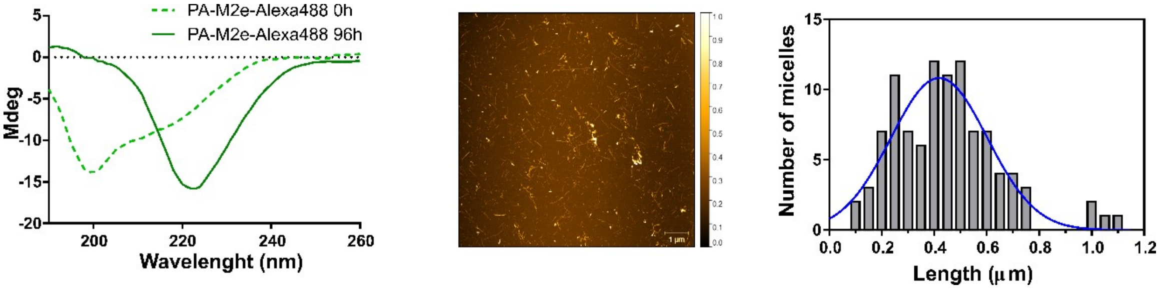

Figure S6 CD spectra, representative of atomic force microscopy and size distribution of PA-M2e-alexa488. Assemblies were obtained after 96h incubation under constant agitation at RT. For the AFM image, scale bar is 1 µm.

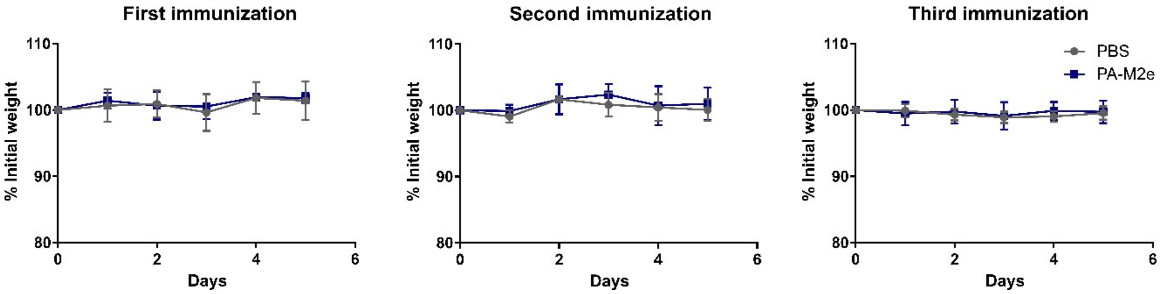

Figure S7 Weight loss of mice after each immunization with PA-M2e and the vehicle control PBS.

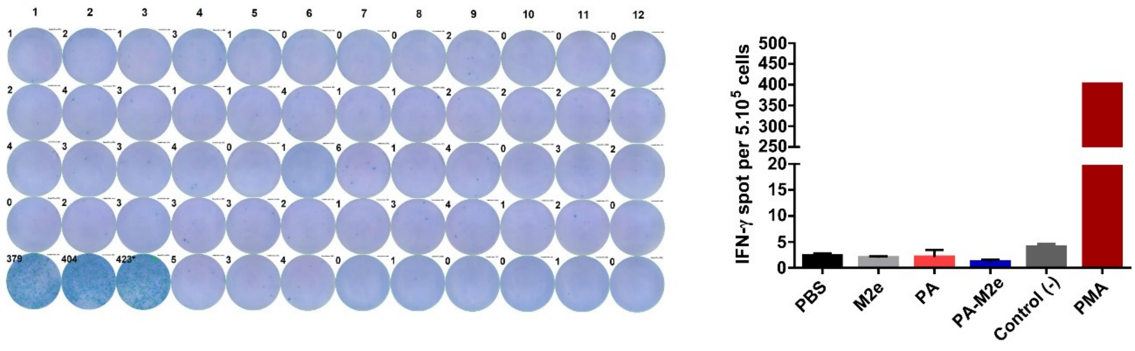

Figure S8 Elispot IFN-γ. ELISpot analysis of IFN γ from ex vivo splenocytes stimulated for 36 h with 2 μg of M2e peptide.

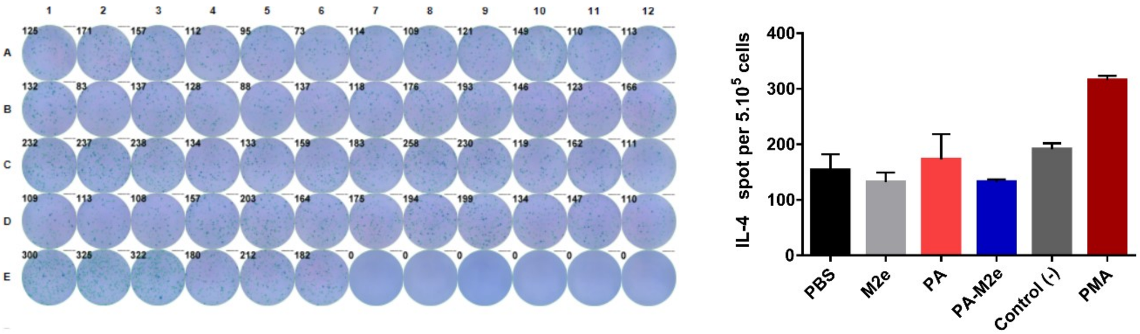

Figure S9 Elispot IL-4. ELISpot analysis of IL-4 from ex vivo splenocytes stimulated for 36 h with 2 μg of M2e peptide.

Table S1. Scale for clinical symptoms of influenza infection

Intensity	Temperature (°C)	Fur	Posture	Eyes	Ears	Response to stimuli	Activity	Feces	Dehydration (Pinch on skin)
**0 (absent)**	> 36	Smooth and even	Normal	Open	Normal	Normal	Normal	Normal	Normal
**1 (light)**	35-36	Fur loss	Slightly hunched back	Half-closed	Bent	Calm, curious	Reduced	Soft	Skin rapidly recovers
**2 (moderate)**	32-35					Delay in response	Immobile, reactive	Sticky	Skin slowly recovers
**3 (severe)**	< 32	Ruffled fur	Hunched back	Closed	Laid down	Inactive	Lethargic	Liquid	No recovery
